# Supplementary figures and images for: Data-driven model discovery and model selection for noisy biological systems
Source: PLoS Comput Biol. 2025 Jan 21;21(1):e1012762. doi: 10.1371/journal.pcbi.1012762 (PMC11753677; doi:10.1371/journal.pcbi.1012762)

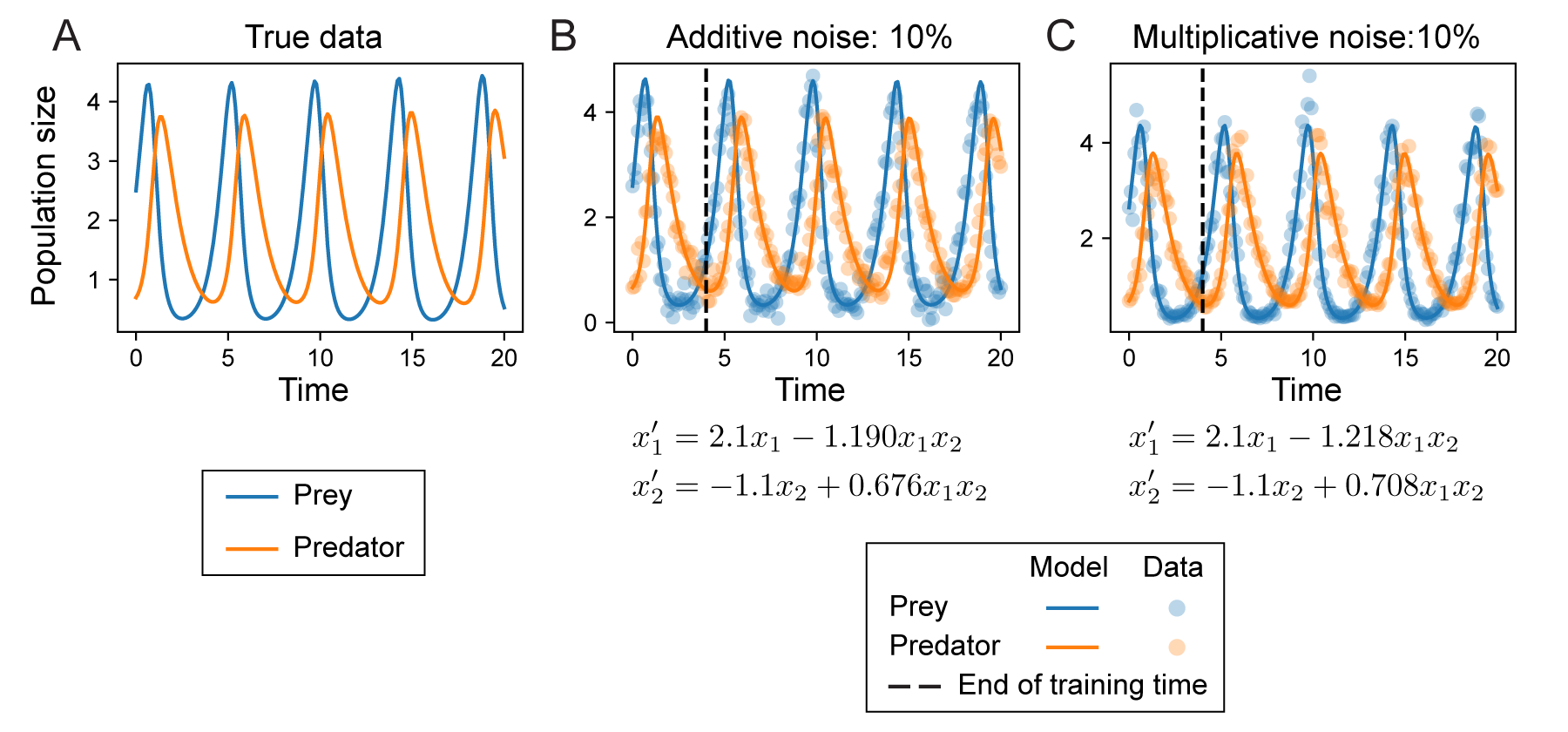

Supplement: S1 Fig — A. Example simulation with parameters { α, β, γ, δ } = { 2 . 1, 1 . 2, 0 . 7, 1 . 1 } . B. Inferred model with lowest AICc for datasets with 10% additive noise. C. Inferred model with lowest AICc for datasets with 10% multiplicative noise. (TIF) [file pcbi.1012762.s001.tif]

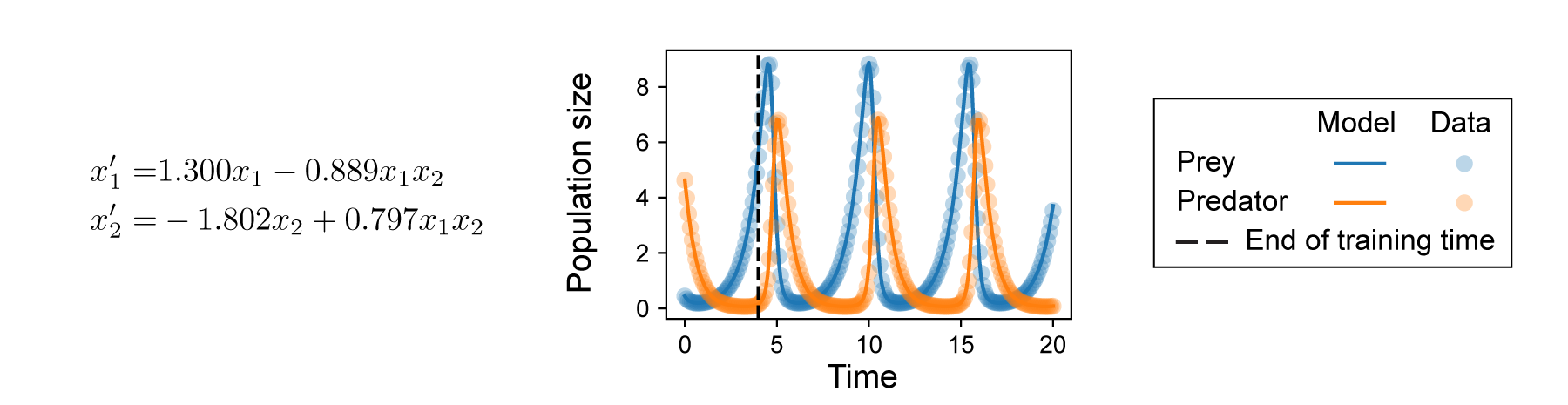

Supplement: S2 Fig — Evaluation of base SINDy to infer Lotka-Volterra models from noise-free data on t = [ 0, 4 ] . True model parameters are { α, β, γ, δ } = { 1 . 3, 0 . 9, 0 . 8, 1 . 8 } ; initial conditions are x0 = [ 0 . 4425, 4 . 6281 ] . Inferred equations (left) and simulation of the inferred model on t = [ 0, 20 ] (right). (TIF) [file pcbi.1012762.s002.tif]

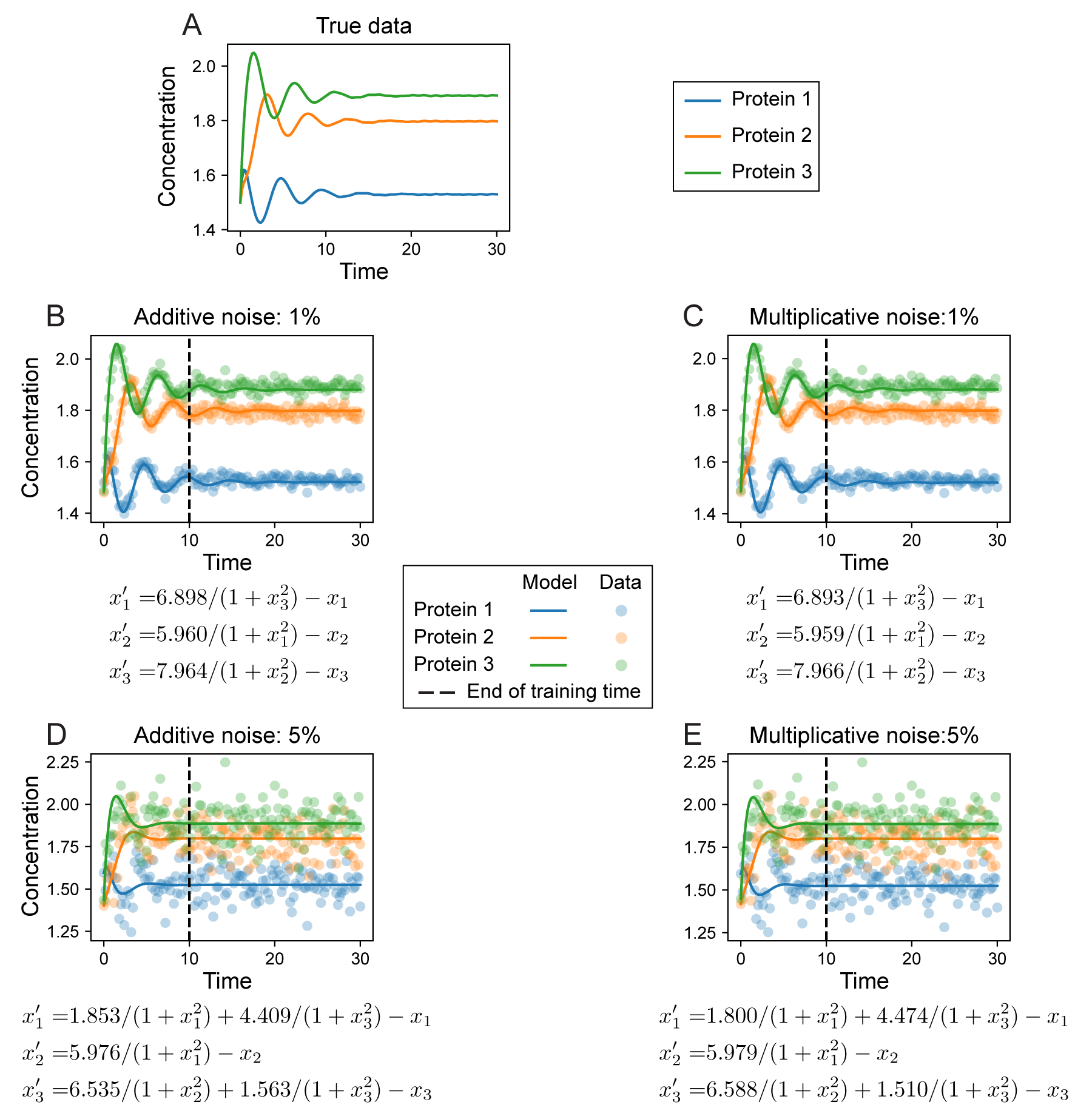

Supplement: S3 Fig — A. Example simulation with parameters β = (7, 6, 8) and n = 2. B. Inferred model with lowest AICc for 1% additive noise dataset. C. Inferred model with lowest AICc for 1% multiplicative noise dataset. D. Inferred model with lowest AICc for 5% additive noise dataset. E. Inferred model with lowest AICc for 5% multiplicative noise dataset. (TIF) [file pcbi.1012762.s003.tif]

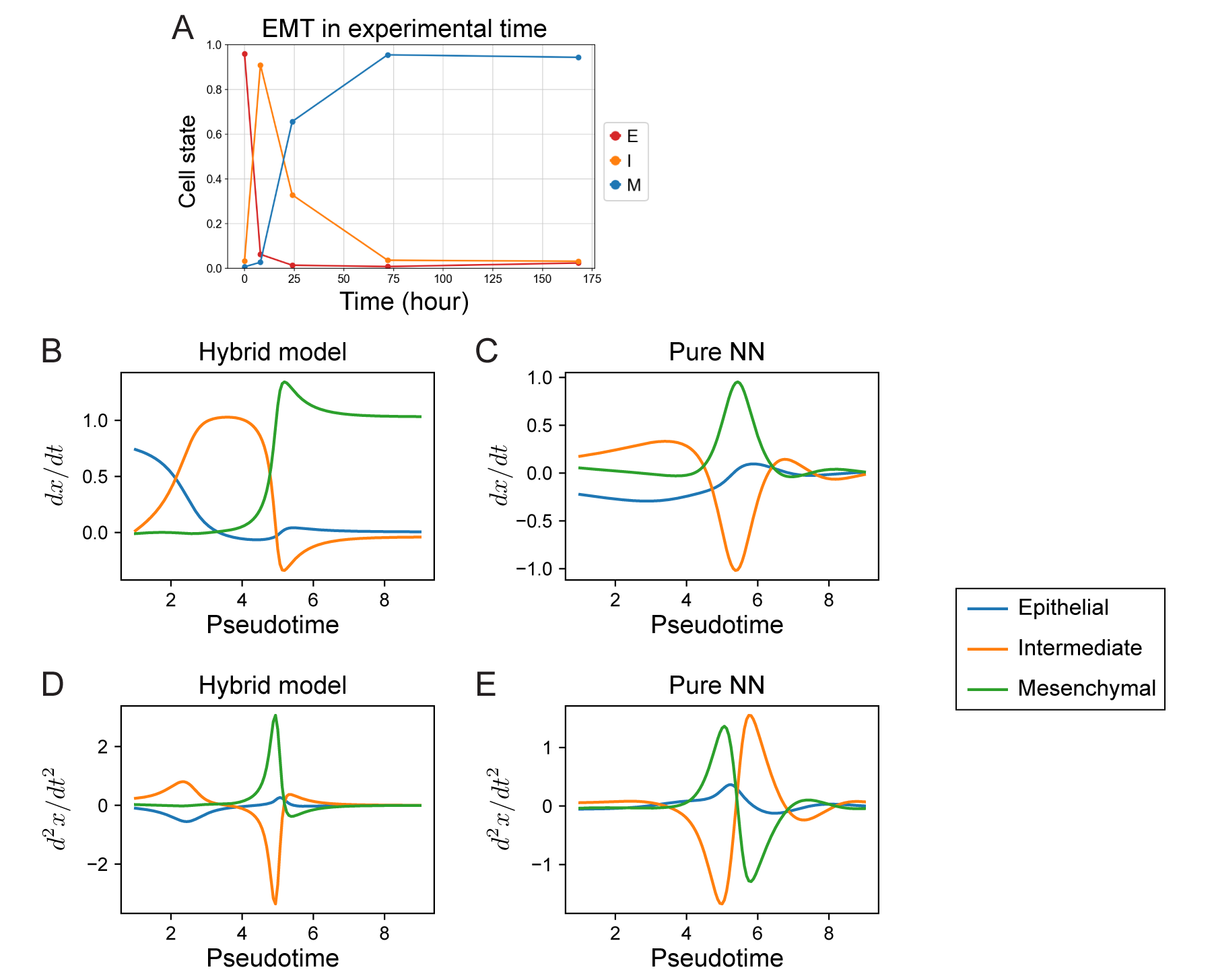

Supplement: S4 Fig — A. Cell state proportions in epithelial-mesenchymal transition in experimental time. B. Derivatives estimated by the trained neural network NN (x) in hybrid formulation (Eqs 9). C. Derivatives estimated by the trained neural network NN (x) in pure neural network formulation (i.e. x′ = NN (x)). D. Derivatives of latent dynamics in A. E. Derivatives of latent dynamics in B. (TIF) [file pcbi.1012762.s004.tif]

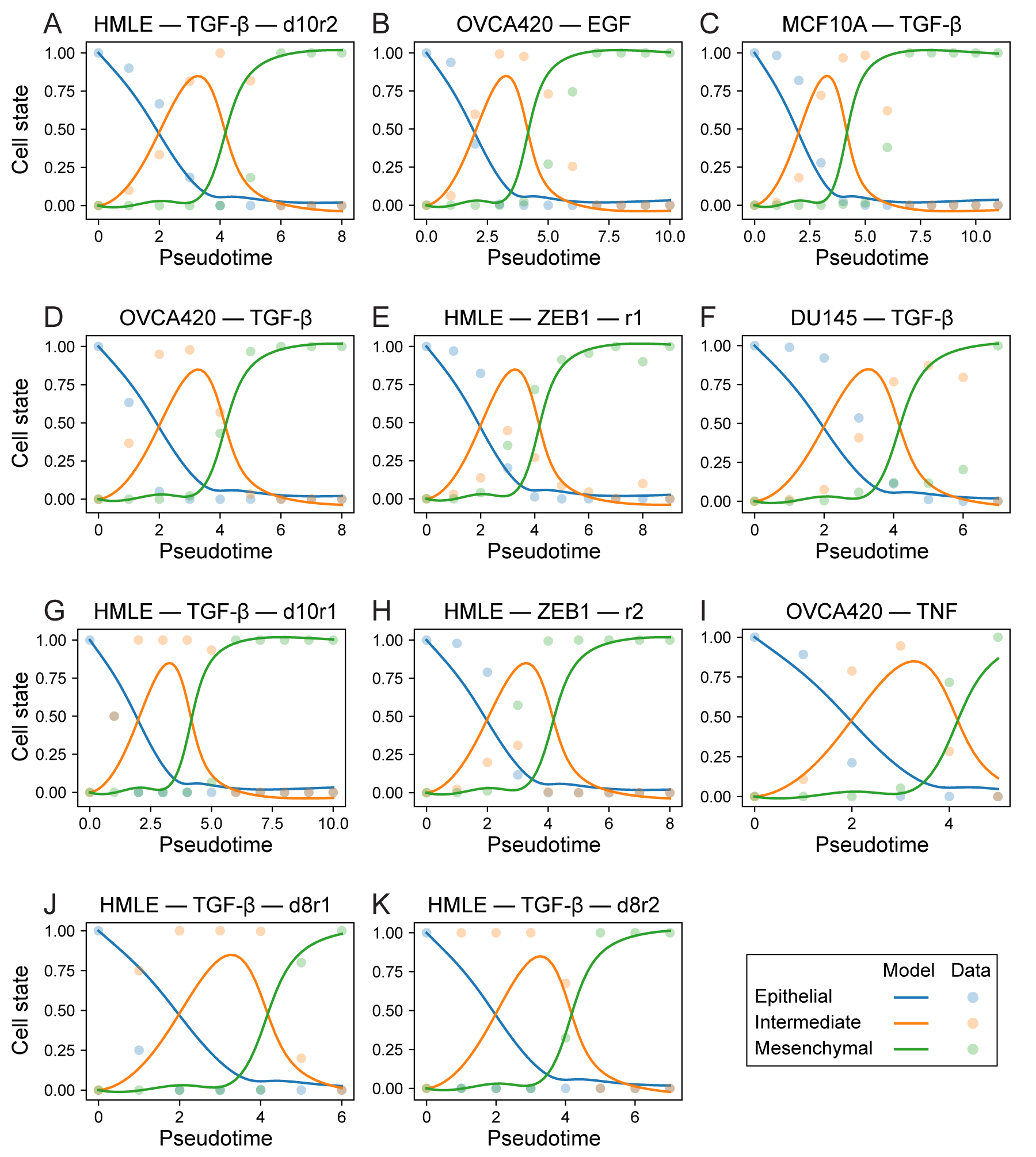

Supplement: S5 Fig — Samples are sorted from smallest mean squared error (A) to largest (K). Each sample is named by “cell line – stimuli”, with label for replicate appended if applicable. The cell lines are: HMLE – human mammary epithelial cell, OVCA – human ovarian cancer cell, MCF10A – human mammary epithelial cell, and DU145 – human prostate cancer cell. For replicates, “d8” and “d10” mean 8 days and 10 days after stimulation, respectively; “r1” means replicate 1 and “r2” replicate 2. (TIF) [file pcbi.1012762.s005.tif]
